# Biological movement and the encoding of its motion and orientation

**DOI:** 10.1038/srep22393

**Published:** 2016-02-29

**Authors:** Christopher P. Benton, Martin Thirkettle, Nicholas E. Scott-Samuel

**Affiliations:** 1 School of Experimental Psychology, University of Bristol, 12a Priory Road, Bristol, BS8 1TU, UK; 2 Faculty of Social Sciences, The Open University, Walton Hall, Milton Keynes, MK7 6AA, UK

## Abstract

Are you walking at me? Biological movement and the encoding of its motion and orientation. A person’s motion conveys a wealth of information that ranges from the complex, such as intention or emotional state, to the simple, such as direction of locomotion. How we recognise and recover people’s motion is addressed by models of biological motion processing. Single channel models propose that this occurs through the operation of form template neurons which respond to viewpoint dependent snapshots of posture. More controversially, a dual channel approach proposes a second stream containing motion template neurons sensitive to view dependent snapshots of biological movement’s characteristic local velocity field. We used behavioural adaptation to look for the co-encoding of viewpoint and walker motion, a hallmark of motion template analysis. We show that opposite viewpoint aftereffects can simultaneously be induced for forwards and reversed walkers. This demonstrates that distinct populations of neurons encode forwards and reversed walking. To account for such aftereffects, these units must either be able to inhibit viewpoint-encoding neurons, or they must encode viewpoint directly. Whereas current single channel models would need extending to incorporate these characteristics, the idea that walker motion is encoded directly, such that viewpoint and motion are intrinsically interlinked, is a fundamental component of the dual channel model.

Biological motion, the pattern and class of articulated motion that is characteristic of animals and humans, plays a central role in our perception of other actors, underpinning our performance on a range of tasks from predation to an understanding of other’s physical and mental intentions. Models of biological motion processing encapsulate the current understanding of how our neural systems extract the relevant information from this intriguing stimulus. Amongst such models, there is general agreement that our recovery of biological motion involves the operation of a processing channel which relies initially on an analysis of form^1^,^2^. Less widely accepted is the idea that we employ an additional parallel channel which acts directly upon stimulus motion^3^,^4^.

Neurons in the form channel are seen to respond to viewpoint dependent snapshots of posture. It is this that determines our perception of body orientation; recognition of direction of motion (forwards/backwards) is then extracted by analysing the sequence of activation of the form snapshot neurons. In contrast, the proposed motion pathway is taken to contain motion template neurons sensitive to view dependent snapshots of biological movement’s characteristic local velocity field. This latter refers to the dense map of motion vectors that results from the application of biological models of motion processing to an image sequence^5^,^6^,^7^. A direct prediction, from the idea of a motion templates for the analysis of biological motion, is that we have neurons that encode both the direction of motion and the orientation of the stimulus relative to the observer. This is because both of these factors clearly determine the pattern of local velocities projected onto an observer’s retinae.

In the current study, we used behavioural adaptation to look for the co-encoding of viewpoint and walker motion. In adaptation, prolonged or repeated viewing of an adapting stimulus affects the perception of a subsequent test stimulus, this change in perception being termed the aftereffect. The premise underlying adaptation experiments is that adaptation to a particular property taps into the neural processes encoding that property. This is because adaptation is seen to be functional, its purpose being to optimise the use of our neural mechanisms to better encode our perceptual input^8^,^9^.

We show that opposite shifts of perceived orientation can simultaneously be induced for forwards and reversed walkers: in other words, our orientation aftereffect was contingent upon whether our walker walked forwards or backwards. Such aftereffects are taken to demonstrate that distinct populations of neurons encode the property upon which the aftereffect is contingent. So, for example, figural aftereffects contingent upon face orientation have been used to demonstrate separate neural populations for the encoding of upright and inverted faces^10^. The logic here is that a single neuron cannot simultaneously be in two different states, so the simultaneous different aftereffects indicate separate neural populations. Our finding therefore demonstrates that separate populations of neurons encode forwards and reversed walking. This, in turn, has direct implications for our current understanding of models of biological motion processing.

## Method

We used motion captured human gait cycle data to animate a computer-generated mannequin. We used a mannequin as this avoided the depth ambiguity found with the more usual point light walker^11^. Additionally, we wrapped our mannequin (henceforth walker) in a simple high contrast texture in order to further reduce form ambiguities. Our walker was presented without translation across the ground plane (treadmill walking) in an orthographic projection from the camera, and animation sequences for each viewpoint angle in a −90° to 90° arc around the mannequin were generated – where 0° indicates the walker directly faced the observer (objectively), and positive angles indicate leftwards rotation. Walker motion, which was either forwards or reversed was manipulated by simply reversing frame presentation order.

**Figure 1:**
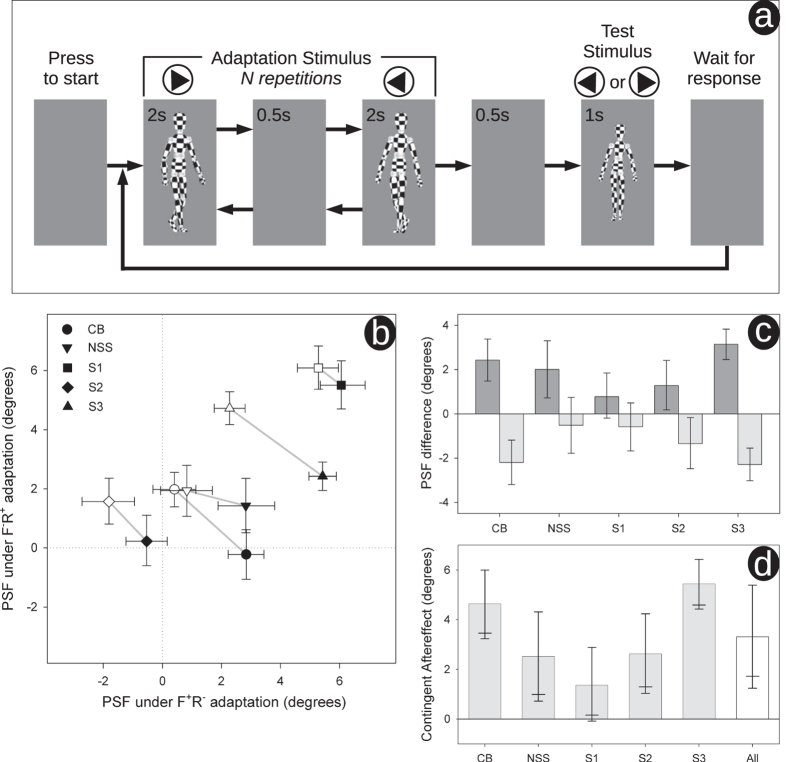
(**a**) schematic diagram of our task. Results are shown in (**b–d**) – see text for description. PSFs were estimated by probit analysis. Observers denoted by initials show authors, remainder are naïve volunteers. All error bars show 95% confidence limits except for (**d**) where the lower 90% are also shown (longer horizontal) as here we have a clear directional prediction. Results collapsed across observers are shown in (**d**) denoted by “All”. Here error bars are derived from the t distribution (t(4) = 4.43, p = 0.01), elsewhere errors bars were determined using bootstrapping.

We used a standard psychophysical method^12^ to determine the viewpoint at which forwards and reversed test walkers appeared to face each observer: the point of subjective facing (PSF). The study was conducted in accordance with the Declaration of Helsinki, and had ethical approval from the Faculty of Science ethics committee at the University of Bristol; informed consent was gained from all research participants. Observers adapted simultaneously to +25° forwards motion and − 25° reversed motion (F + R−), being shown short segments of each interleaved together (see Fig. 1a). In separate blocks the same observers adapted simultaneously to −25° forwards motion and +25° reversed motion (F−R +).

Observers each completed 4 runs of 80 trials under each simultaneous adaptation condition. Each trial consisted of adaptation followed by a test stimulus. In the first trial, adaptation consisted of 24 repetitions of two adaptors regularly alternating; all adaptors were separated by a 0.5 s blank screen (48 s per adaptor). Adaptation in subsequent trials (top-ups) consisted of 2 repetitions. The test stimulus was either forwards or reversed (40 of each per run), order randomised. Each walker segment was chosen at random from a longer sequence (10 s for adaptors, 5 s for tests). Gait cycle was ~1.36 s (motion captured from author MT). An example movie sequence, showing two top-up trials may be found in our supplementary material.

To minimise low-level retinotopic adaptation, on-screen adaptors were 25% larger than test stimuli^13^. Viewing distance was ~100 cm, adaptor height was 317 pixels (~6.8°), test height was 253 pixels (~5.4°). Stimuli were presented on a linearised Lacie electron blue IV monitor (1024 × 768 pixels, 75 Hz, mean luminance 61 cd/m^2^).

## Results

Repeated activation typically reduces neural activity; consequently, adaptation biases responses away from the adaptor^14^. Adaptation to a rightwards facing adaptor therefore leads to a stimulus that would otherwise be judged as directly facing the observer to appear oriented to the left. So in order to now be judged as directly facing, the test stimulus needs to be rotated rightwards (towards the adaptor). Essentially, the standard repulsive effect of adaptation is counter intuitively indexed by a shift of the PSF towards the adaptor. Under F + R− adaptation, if there is a contingent aftereffect, we would expect the PSF of the forwards test to shift positively, whilst that of the reversed test should shift negatively (and vice versa for F−R + adaptation).

Figure 1b plots the PSFs for the forwards (filled symbols) and reversed (open symbols) test stimuli under the two simultaneous adaptation conditions. PSFs were estimated by fitting cumulative normal distributions to our psychometic data^15^ resulting in two psychometric functions per run (one for forward test stimuli, one for reversed). To assess statistical variability^16^, we used parametric bootstrapping, generating 10000 bootstrap estimates per PSF^17^. We then propagated the bootstrap populations through the relevant averaging and differencing calculations described below to generate our plotted confidence limits^18^. These were calculated using the percentile method^19^.

Compared to the results for the forwards test sequences, results for the reversed are shifted leftwards and upwards. This indicates that under F + R− adaptation (when compared to F−R + adaptation) the PSF for the forwards walking test walker is shifted towards the viewpoint of the forwards walker adaptation walker (F+). Conversely, the PSF of the reversed test is shifted towards the viewpoint of the reversed adaptor (R−). This is made clear in Fig. 1c where we plot the PSF difference between forwards and reversed test walkers under F + R− adaptation (dark bars) and under F−R + adaptation (light bars). The opposite pattern of shifts under the different simultaneous adaptation conditions become self-evident. Figure 1d summarises these opposite shifts by taking the difference to arrive at an overall metric of the contingent aftereffect.

**Figure 2:**
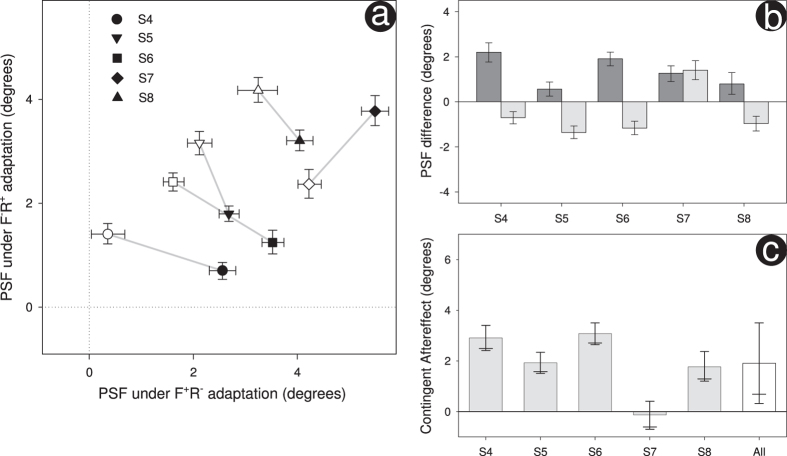
Results for the repeat of our initial experiment are shown in (**a–c**) – these graphs mirror those shown in Fig. 1b–d. As before, all error bars show 95% confidence limits except for (**c**) where the lower 90% are also shown (longer horizontal). Results collapsed across our 5 additional observers are shown in (**c**) denoted by “All”. Here error bars are derived from the t distribution (t(4) = 3.33, p = 0.03), elsewhere errors bars were determined using bootstrapping.

The results that we describe above are based on the responses drawn from two authors and three naïve observers. At the prompting of an anonymous reviewer, we repeated our experiment with 5 additional naïves. To determine the required number of observers we performed a power calculation (1−β = 0.9, α = 0.05) using the contingent adaptation shown by our three naïves (μ = 3.14°, σ = 2.09°) to estimate effect size. For this repetition, all stimuli were displayed on an Iiyama Vision Master Pro 513 monitor (mean luminance 53 cd/m^2^); all other experimental details were the same. Results are shown in Fig. 2. As with each experiment analysed individually, we find significant contingent adaptation when collapsed across experiments (t(9) = 5.20, p < 0.001).

## Discussion

Our results show a clear contingent aftereffect with the perceived orientation of a walker being dependent upon whether the motion of that walker is forwards or reversed. This is evident when collapsed across participants and is generally seen on a participant-by-participant basis (apart from one observer who appears somewhat atypical-see S7 in Fig. 2). This contingent aftereffect can readily be explained by the notion of motion template neurons. Such neurons are necessarily viewpoint-selective because the local image motions to which they respond are viewpoint dependent. Separate viewpoint adaptation would take place within those neurons sensitive to forwards motion and those sensitive to reversed motion, thereby producing our sequence-contingent viewpoint aftereffect.

Whilst the above provides potentially the most straightforward account, we should also consider whether a form processing stream can reasonably account for our findings. In descriptions of form processing channels, walker orientation and walker sequence direction are processed at different stages: the former is drawn from the population of orientation selective form-template neurons, the latter is determined by second-stage units sensitive to the order of form template neuron activation^1^,^2^. This arrangement could account for sequence dependent viewpoint adaptation by incorporating inhibitory connections running between second- and first-stage units, with inhibition increasing with correlation. Repeated presentation of a forwards walker, at a particular orientation, would lead to a reduction in neural activity of units tuned to that orientation; but only in response to a forwards walker. Combining this with adaptation to a reversed walker at a different orientation gives us a mechanism that can generate different viewpoint aftereffects contingent upon walker sequence direction.

In the dual channel model’s account of the operation of its form channel^3^,^20^, sequence direction is encoded through asymmetric connections between form template neurons. We can reasonably ask whether this mechanism could, by itself, provide an account of our findings. When a particular form template is excited, it preferentially excites the next form template neuron in the sequence, whilst inhibiting the previous one. These connections arise through Hebbian learning so that the population of viewpoint-tuned form template neurons develops *increased* activity in response to commonly seen motions (rather than the *reduced* activity found with adaptation)^21^. So while this putative form channel does contain neural structures sensitive to both orientation and sequence direction, it predicts an attractive effect of adaptation, opposite to that observed.

As currently described, proposals for the form-based analysis of biological motion do not account for our motion-contingent orientation aftereffect. Whilst such approaches can undoubtedly be extended, the idea of motion template neurons, drawn directly from the idea of a motion processing stream for biological motion, provides a ready and straightforward explanation of our finding. This point is strengthened by recent convincing evidence for separate form and motion channels running, respectively, through the extrastriate body area and posterior superior temporal sulcus^22^. This support for a two channel architecture neatly dovetails with our confirmation of a prediction drawn from a critical algorithmic component of that viewpoint: namely, the motion template analysis of biological movement.

## Additional Information

**How to cite this article**: Benton, C. P. *et al*. Biological movement and the encoding of its motion and orientation. *Sci. Rep.* **6**, 22393; doi: 10.1038/srep22393 (2016).
